# Synthesis of Pluronic F127 copolymer/iron oxide–GelMA nanocomposite for doxorubicin drug delivery

**DOI:** 10.1039/d5na00776c

**Published:** 2026-01-02

**Authors:** Reza Davarnejad, Kimia Haghighatnejad, Omid Sartipzadeh Hematabad, Zahra Mohammadpour, Majid Komijani, John F. Kennedy

**Affiliations:** a Department of Chemical Engineering, Faculty of Engineering, Arak University Arak 38156-8-8349 Iran r-davarnejad@araku.ac.ir rdavarne@uwo.ca; b Nanobiotechnology Group, Multi-disciplinary and Interdisciplinary Department, Arak University Arak 38156-8-8349 Iran; c Biomaterials and Tissue Engineering Research Group, Department of Interdisciplinary Technologies, Breast Cancer Research Center, Motamed Cancer Institute, ACECR Tehran 15179-64311 Iran; d Medical Nanotechnology Department, Breast Cancer Research Center, Motamed Cancer Institute, ACECR Tehran 1517964311 Iran mohammadpour@acecr.ac.ir + 98-86-34173450 +98-9188621773; e Department of Biology, Faculty of Science, Arak University Arak 38156-8-8349 Iran; f Chembiotech Laboratories Kyrewood House, Tenbury Wells WR15 8SG Worcester UK

## Abstract

In this study, a drug polymeric nanocarrier system consisting of Pluronic F127 copolymer/GelMA iron oxide nanoparticles was prepared to load and release doxorubicin. Various weight ratios of the polymer were tested to determine the highest drug entrapment rate and the optimal size of the composite. Results showed that the maximum rate of drug entrapment in the system was 57%. GelMA was synthesized and analyzed by FTIR and FESEM. Various weight ratios of gel were tested to determine an optimal concentration. The swelling rate and degradability of hydrogels were evaluated. It was found that GelMA with a concentration of 10% had more swelling and degradability. Therefore, it was chosen as the optimal concentration. Finally, the drug system was investigated using FTIR spectroscopy, FESEM, XRD, TGA and VSM. Results showed that the drug delivery system exhibited slow release and followed the Korsmeyer–Peppas mechanism.

## Abstract 2

A drug polymeric nanocarrier system consisting of Pluronic F127 copolymer/GelMA iron oxide nanoparticles was prepared to load and release doxorubicin. It was found that GelMA with a concentration of 10% exhibited more swelling and degradability.

## Graphical abstract

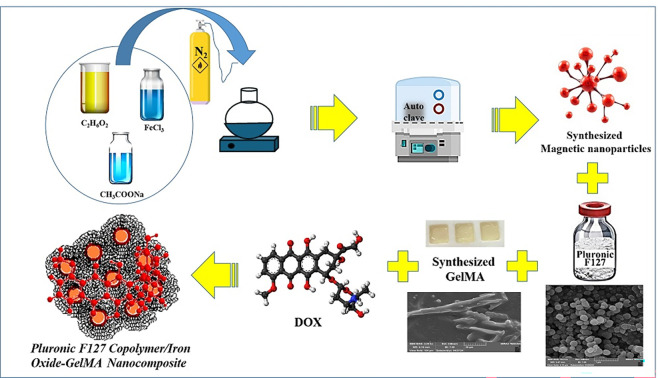

## 1. Introduction

Various methods have been used to treat cancer, such as surgery, radiotherapy and chemotherapy.^1^ However, the success of chemotherapy, one of the most important methods, depends on the drug delivery system and the type of cancerous tumor.^2,3^ The favorable characteristics of drug delivery systems, such as natural access, controlled release and low toxicity effects, have increased their applications.^4^ An ideal drug delivery system should load the drug and precisely transfer it to the target organ. Drug nanocarriers allow for continuous and controlled drug release while maintaining the drug level. They interact on the surface and inside the biological cells. They may deeply penetrate a tissue due to their small size.^5^

Alexandridis *et al.* found that micelle formation is thermodynamically a positive entropy process.^6^ Linse discussed the critical micelle concentration (CMC), critical micelle formation temperature (CMT), aggregation number and different hydrodynamic radii of the Pluronic block copolymers in aqueous solutions.^7^ Shen *et al.* investigated the controlled release of doxorubicin (DOX) loaded on Zein.^8^ Shaikh *et al.* considered the drug entrapment, release, and retention efficiency in terms of the transition metal ion content and morphology of doxorubicin in the liposomal system and its therapeutic potential.^9^

Mosafer *et al.* conjugated doxorubicin-carrying PLGA-coated SPIONs to the AS1411 aptamer. They successfully tested it on the intestinal carcinoma cells of various categories of mice.^10,11^ Alyane *et al.* prepared nanoliposomes containing doxorubicin using a gradient method.^12^ They used nanoliposomes with a size of 100 nm and achieved around 90% drug entrapment. Haghiralsadat *et al.* loaded doxorubicin into liposomal carriers to treat bone cancer.^13^ They produced nanosystems with a size of 126 nm, achieving an encapsulation efficiency of 89%. The maximum release of doxorubicin was around 46% for 48 h. Saravanakumar *et al.* successfully delivered doxorubicin to a cancer cell line using polylactic-glycolic acid nanoparticles attached to the AS1411 aptamer.^14^

Pluronic F127 micelles, GelMA hydrogels and iron oxide nanoparticles have each been extensively investigated for drug delivery due to their unique properties such as thermoresponsiveness, biocompatibility and magnetic guidance. However, these systems show some limitations in drug loading efficiency, controlled release and multifunctionality when they are used individually.^15^

Carrera Espinoza *et al.* successfully delivered doxorubicin to a cancer cell line using smart supermagnetic nanocomposites based on iron oxide nanoparticles coated with Pluronic F127.^16^ They increased the drug release in acidic pH, which may be due to the pH sensitivity of the polymer. According to the *in vitro* results, a survival rate of 90% in HepG2 cells treated with the nanocomposite was observed. Furthermore, the synthesized smart nanocomposite showed drug delivery to liver cancer, overcoming the limitations of traditional therapies.

Herein, a composite system combining Pluronic F127, GelMA and iron oxide nanoparticles, which leverages the synergistic advantages of each component, was prepared and applied. The thermoresponsive Pluronic F127 provides enhanced drug entrapment and release control, GelMA hydrogels offer a tunable and biocompatible network, and iron oxide nanoparticles introduce magnetic responsiveness for good potential guided delivery.^17^

According to the literature on the doxorubicin carriers such as liposomes or polymeric nanoparticles, it seems that the hybrid system proposed in this research can show better characteristics such as higher drug loading efficiency, tunable particle size and pH-responsive release.^15^ Likewise, a system based on the Pluronic F127 copolymer/iron oxide–gelatin methacrylate (GelMA) nanocomposite for drug delivery was made. Various analytical techniques, such as FTIR, FESEM, XRD, TGA and VSM, were used to investigate the physicochemical properties of the doxorubicin medicinal system, and then, the kinetics of doxorubicin release was investigated in an aqueous environment.

Therefore, the aim of this study is to develop an optimized Pluronic F127/GelMA/iron oxide nanoparticle nanocarrier with enhanced drug encapsulation, controlled release behavior and good potential for improved therapeutic performance.

## 2. Materials and methods

Doxorubicin–HCl (DOX) [C_27_H_29_NO_11_·HCl, 98–102%, (50 mg/25 ml)] was purchased from EBEWE Pharma Co. Pluronic^®^ F-127 (PF) [(C_3_H_6_O·C_2_H_4_O)_*x*_, Bio-Reagent], sodium acetate (CH_3_COONa, ≥99.0%), ethylene glycol (C_2_H_6_O_2_, ≥99.0%), and ferric chloride hexahydrate (FeCl_3_·6H_2_O, 98%) were used as the precursors and precipitating agent of the coated magnetic nanoparticles. Gelatin (Bovine Skin, Type B), methacrylic anhydride (MA) (C_8_H_10_O_3_, ≥98.0%), ethylenediaminetetraacetic acid (EDTA) (C_10_H_16_N_2_O_8_, ≥98.0%), sodium bicarbonate (NaHCO_3_, 99.5%), and phosphate-buffered saline (PBS, pH = 7.4) were applied in GelMA synthesis. 2-Hydroxy-4′-(2-hydroxyethoxy)-2-methylpropiophenone (Irgacure2959, 98.0%), diphenyl(2,4,6-trimethylbenzoyl) phosphine oxide (TPO, 97.0%), methanol (MetOH, 99.0%), and ethanol (EtOH, 96.0%) were utilized as the photoinitiator, dissolving and washing agent, respectively.

### 2.1. Synthesis of GelMA

A tablet of PBS was dissolved in 500 ml of deionized (DI) water to set the pH at 7.4. Next, 100 ml of the above solution was mixed with 10 g of gelatin powder and stirred with a magnetic stirrer at 400 rpm at a temperature of 60 °C for 2 h. Then, 10 ml of MA was dropwise added to the solution with a syringe pump. The solution temperature was then reduced to 55 °C and stirred for 3 h. The solution was diluted with DI with a ratio of 1/4 (v/v) to stop the reaction and was continuously stirred for 1 h. The synthesized Gelatin Methacryloyl (GelMA) was added to the dialysis tubing. For dialysis bag preparation, 0.84 g of sodium bicarbonate and 0.372 g of EDTA were dissolved in 200 ml of DI water and stirred to obtain a clear and uniform solution. The solution was heated on a stirrer-heater to boil. The dialysis bag was added to the boiling solution. The dialysis bag was boiled for 30 min and finally washed with DI water to remove the excess material.^18^ The solution of synthesized GelMA was added to the dialysis bag and kept in the oven at 45 °C for one week. The safe handling, storage and disposal of methacrylate anhydride have been shown in Fig. S1 (SI Section).

A dialysis bag (with a cut-off of 14 kDa) was used to remove possible residual toxic impurities such as methacrylate anhydride, by-products and unreacted monomers. Since all undesirable substances in the dialysis bag, except GelMA, have a molecular size smaller than the size of the dialysis bag, they diffuse out of the bag due to the concentration difference (inside and outside the bag) when the bag is placed in DI water. The water in the dialysis chamber was replaced every 6 h to increase GelMA purification from undesirable substances. The material was emptied from the dialysis bag after 7 days and freeze-dried to obtain a uniform powder of GelMA.

### 2.2. Swelling index of GelMA

Three different concentrations of GelMA, 10, 15, and 20% (w/v), were prepared by adding GelMA powder to 5 ml PBS (pH = 7.4) and stirring for 15 min at 45 °C until a homogeneous solution was obtained.^19^ Then, 20 mg of photo-initiator TPO was dissolved in 0.5 ml of MeOH and placed on the stirrer, and 20 mg of photo-initiator Irgacure was added to the TPO solution to obtain a uniform solution. Afterwards, 0.1% w/w of the photo-initiator solution was added to each of the GelMA solutions with different concentrations. Finally, the solution was irradiated under UV light (250 W, NOOR, Iran) in a rectangular cube mould made of PTFE with dimensions of 12 × 12 × 4 mm^3^ for 20 min to form the gel.

The samples obtained from the previous stage were initially weighed and then immersed in 15 ml of PBS solution (pH = 7.4). The lids of the beakers were tightened with aluminum foil, and the beakers were placed in an incubator at 37 °C for 48 h. The samples were then taken out, filtered, and weighed, and placed in a 37 °C incubator for 10 min to evaporate the surface liquid on the GelMA, then weighed again. The swelling capacity of the gel was determined as follows:1
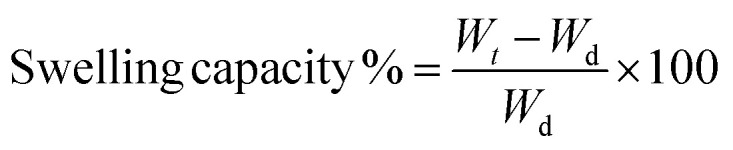
where *W*_*t*_ and *W*_d_ are the weights of the hydrated sample at time *t* and of initial dry sample, respectively.

### 2.3. Degradation index of GelMA

Various concentrations of GelMA were first poured and cured in the mould, followed by weighing and placing them into 10 ml of PBS solution. According to the static incubation conditions, the containers were tightly sealed with aluminum foil and placed in an incubator at 37 °C. The sampling process was carried out as it was on the first day, where 200 µl of the solution from each container was removed and transferred to 0.5 ml microtubes and then replaced with 200 µl of fresh PBS solution. This process was repeated daily for 90 days. Finally, the contents of the containers were centrifuged at 4000 rpm for 10 min at room temperature (25 °C ± 1 °C). The supernatant was discarded, and the residue was washed three times with DI water. The remaining solid was dried overnight in an oven at 37 °C, weighed, and the degradation percentage of each sample was calculated using the following equation:2
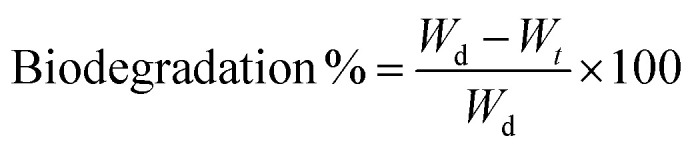
where *W*_d_ and *W*_*t*_ are the weights of the initial dry sample and sample at time *t*, respectively.

### 2.4. Synthesis of PF-coated magnetic nanoparticles (MNPs)

Here, 1.23 g of FeCl_3_·6H_2_O was poured into a two-necked flask. Then, 75 ml of ethylene glycol was added to the flask and stirred to obtain a uniform solution under neutral gas (N_2_). Then, 3.66 g of dry sodium acetate was added to the solution and stirred vigorously with a mechanical stirrer for 1 h. Subsequently, the yellowish solution was heated in a Teflon-lined stainless-steel autoclave at 200 °C for 16 h. The solution was removed from the autoclave and poured into a beaker, and washed several times with DI water and EtOH. The final products were dried in a vacuum oven overnight at 37 °C. PF was dispersed by sonication in DI water for 30 min to produce a uniform solution with various concentrations, 1%, 2% and 5% w/w. The surface-modified MNPs with PF were formulated using three different concentrations of polymer, mixed with 100 mg of MNPs, and stirred overnight. The MNPs coated with PF were washed numerous times with EtOH and DI water. Finally, the nanocomposites (MNPs-PF-1, MNPs-PF-2, and MNPs-PF-5) were dried at 65 °C overnight.^16^

### 2.5. Preparation of DOX-loaded MNPs-PF

Here, 6 mg of each sample (MNPs-PF-1, MNPs-PF-2, and MNPs-PF-5) was separately mixed with 1 mg of DOX dissolved in 1 ml of water for injection. The solution had a natural pH of approximately 5.5 (within the range of 5.0–7.0), and no additional pH adjustment was performed to maintain DOX stability. The mixture was kept under constant shaking in a dark environment at room temperature for 48 h. The suspension was centrifuged at 2500 rpm for 5 min, and the supernatant solution was removed at the end of the loading period. The precipitate (DOX-loaded nanoparticles) was obtained by centrifugation at 13 000 rpm for 10 min, and the acquired pellet was placed in an oven at 65 °C until its weight stabilized.^16^ The drug concentration in the supernatant solution (floating on the surface) was measured using a UV-Vis spectrophotometer (PerkinElmer America, Lambda 25) at the maximum absorption wavelength of 481 nm. The calibration curve of doxorubicin absorption *versus* its concentration was obtained. The drug-loading efficiency (DLE) and entrapment efficiency (EE) of DOX-loaded in the composites (nanoparticles) were respectively calculated using the following:3
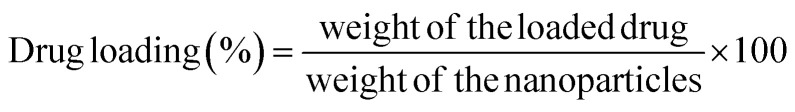
4
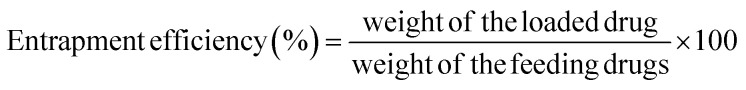


### 2.6. Preparation of the DOX-loaded MNP-PF/GelMA composite

DOX-loaded MNPs-PF/GelMA nanocomposites were prepared at three distinct weight percentages involving 10%, 15%, and 20% GelMA. Here, 0.2 g of GelMA powder was added to 2 ml DI water and stirred till a homogeneous solution was obtained. Then, 0.1% (w/w) of the photoinitiator solution [(relative to GelMA) prepared in Section 2.2] was added to the solution and stirred for 10 min in the dark.

Next, 5 mg of DOX-loaded MNPs-PF (as prepared in Section 2.5) of each percentage of PF was added to GelMA with cross-linker agent and stirred vigorously for 30 min in a dark environment. Finally, the entire mixture was added to the PTFE mould and placed under a UV lamp at a distance of 10 cm for 20 min for the crosslinking step. GelMA was polymerized under UV light (250 W, UVA 360–405 nm) to form the GelMA hydrogel.

### 2.7. Assessment of drug release *in vitro*

The determination of DOX release from MNPs coated with PF was conducted using the following method.

Here, 6 mg of desiccated DOX-loaded MNPs-PF were transferred to a 5 ml tube, and 2 ml of PBS solution was added to the tube. Subsequently, the tube was positioned on a roller stirrer at 37 °C. The samples were extracted from the solution at various time intervals to quantify the DOX release in the solution. Following each sample, an equivalent volume of new buffer was added to ensure the medium's concentration remained constant. The released drug (DOX) content was quantified by detecting its absorbance at 485 nm using the UV-Vis spectrometer. Calibration curves were generated for the medications using standard methods under controlled experimental circumstances. The medication release (%) was calculated as follows:5
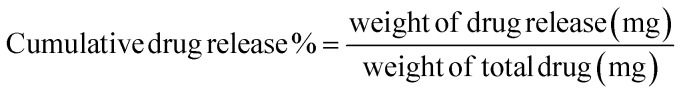


### 2.8. Release mechanism

Mathematical modeling should be very useful because it allows the prediction of the release kinetics. In fact, it would be possible to determine some important parameters, such as the drug diffusion coefficient. Therefore, mathematical modeling requires a sufficient understanding of all phenomena affecting the drug release kinetics. The kinetics of drug release from the carriers depends on some factors such as the geometric structure of the matrix, the type of drug and the drug release mechanism. There are several kinetic release models, such as zero-order,^20^ first-order,^21,22^ Higuchi,^23^ and Korsmeyer–Peppas^24^ for drugs. Several renowned mathematical models were evaluated to investigate the mechanism of DOX release from composites, as detailed below:

• Zero-order model

The zero-order model is used for samples in which the release rate of the drug is independent of the dosage of the soluble medicine. This model is represented as follows:

6 *Q*_*t*_ = *K*_0_*t*

where *t* and *K*_0_ are time and the rate constant of the zero-order model, respectively. *Q*_*t*_ is the cumulative value from the drug released at time *t*.

• First-order model

The first-order model is used for samples where the rate of release depends on drugs like DOX. This is expressed as follows:7
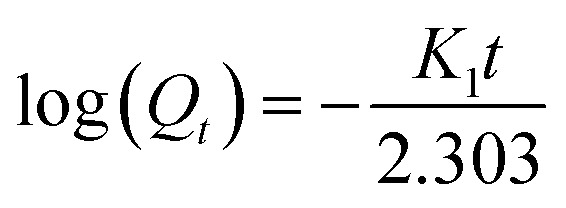
where *K*_1_ and *t* are the first-order velocity constant and time, respectively. *Q*_*t*_ is the cumulative value of the drug remaining at time *t*.

• Higuchi model

The release of a drug depends on the time. In this case, the drug release mechanism depends on Fick's law. The model is represented as follows:

8 *Q*_*t*_ = *K*_H_*t*^0.5^

where *K*_H_ represents the initial constant speed and *t* denotes time. *Q*_*t*_ refers to the accumulated amount of the drug discharged at a specific time *t*.

• Korsmeyer–Peppas release model

The Korsmeyer–Peppas model is utilized to gain insight into the release mechanism of the drug. According to the power law model, this elucidates how drugs are released from polymeric systems. This model is particularly useful for characterizing systems with multiple mechanisms or unclear release processes. This model is represented as follows:

9 *Q*_*t*_ = *K*_KP_ × *t*^*n*^

where *K*_KP_, *t*, and *n* are the Korsmeyer–Peppas rate constant, time, and the release exponent. *Q*_*t*_ is the cumulative amount of drug released at time *t*.

### 2.9. Characterization

Fourier Transform Infrared spectroscopy (FTIR) was used to determine the functional groups and their roles in the adsorption process. Scanning Electron Microscopy (SEM) was used to examine the nanocomposite surface characteristics and morphology. The magnetic behavior of iron oxide nanoparticles and the combination of nanoparticles and polymer were investigated using a Vibrating Sample Magnetometer (VSM). The X-ray Diffraction (XRD) technique was used to determine the crystal structure. Thermogravimetric analysis (TGA) of iron oxide under an argon atmosphere with a heating rate of 10 °C min^−1^ was also conducted in the temperature range of 25–600 °C.

### 2.10. Cell cytotoxicity study

The human breast cancer MCF-7 cell line [provided by the Pasteur Institute of Iran (Tehran)] was grown in DMEM high glucose medium (Gibco) supplemented with 10% fetal bovine serum (FBS) (Gibco) and penicillin/streptomycin 1% (Gibco) in a humidified incubator under a temperature of 37 °C, relative humidity of 90%, and 5% CO_2_. The cells were prepared by washing in PBS when they reached confluence, and were separated from the dish using trypsin–EDTA (Gibco). The cells were then resuspended to achieve a concentration of 1 × 10^4^ cells per ml after being moved to a centrifuge tube and spun for 5 min at 1100 rpm. MCF-7 cells were seeded in a 96-well plate at a density of 1 × 10^3^ cells per well, 24 h before the *in vitro* cytotoxicity studies. MNP formulations coated with PF with 1%, 2%, and 5% concentrations were loaded with DOX prepared with PBS (pH 7.4 and 5.5), as described above. Control formulations without DOX were also prepared using the same procedure. The four stock solutions were filtered through sterile 0.22 µm Millipore paper, and then further diluted with sterile PBS (pH 7.4 and 5.5) step-wise to obtain DOX concentrations in the range of 3.125–200 mg ml^−1^. Here, 100 µl of the DOX formulations and 100 µl of the culture medium (DMEM medium + 10% FBS) were added to the cells to evaluate their cytotoxic effects. As the control, 100 µl of PBS (pH 7.4 and 5.5) was added to the cells, as well as 100 µl of the culture medium. The cells were incubated for 24, 48, and 72 h at 37 °C in a humidified atmosphere with 5% CO_2_. After incubation, the number of viable cells was determined by 3-(4,5-dimethylthiazol-2-yl)-2,5-diphenyltetrazolium bromide (MTT) colorimetric assay.

Here, 20 µl of MTT solution (5 mg ml^−1^) was added to each well. The plates were incubated for an additional 4 h, and then the medium was discarded. A volume of 150 µl of DMSO (dimethyl sulfoxide) was added to each well, and the solution was vigorously mixed to dissolve the reacted dye. The absorbance of each well was read on a microplate reader (BioTek Instruments, ELx800, USA) at a test wavelength of 570 nm and reference wavelength of 650 nm. The samples were tested in triplicate, and six wells containing only culture medium served as blanks. The relative cell viability (%) was calculated as follows:10
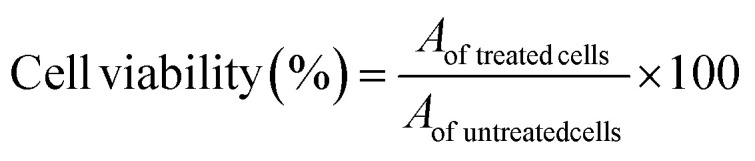


### 2.11. Statistical analyses

Analysis of variance (two-way ANOVA) was applied for statistical analysis. Data were analyzed in triplicate and are presented as mean ± standard deviation (SD). The data obtained at *p* ≤ 0.05 were considered statistically significant.

## 3. Results and discussion

### 3.1. Characterization of MNPs and the DOX-MNPs-PF/GelMA composite

**Fig. 1:**
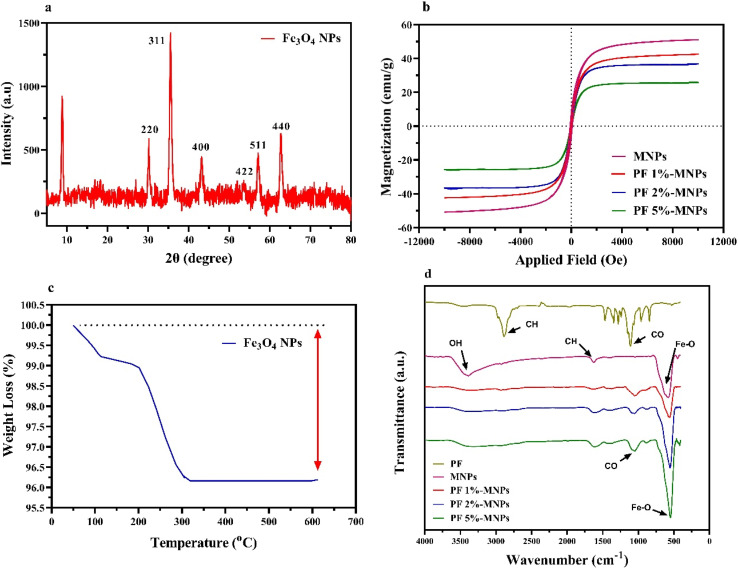
(a) XRD pattern of iron oxide nanoparticles. (b) Magnetization curves of MNPs. (c) TGA analysis curve of MNPs. (d) FTIR spectra of PF, MNPs, and three different concentrations of PF-MNPs.

In order to study the nature of the obtained product, XRD analysis was performed on the MNPs. As shown in Fig. 1a, the specific peaks of Fe_3_O_4_ were observed at 2*θ* values of 30.24°, 35.52°, 43.12°, 53.48°, 57.16° and 32.68°, which correspond to the Miller indices of the reflection plane of (220), (311), (400), (422), (511), (440), respectively.

The Fe_3_O_4_ magnetic nanoparticles form a cubic crystal structure.^18,25^ The magnetic properties of MNPs and the MNPs coated with various percentages of PF (1%, 2%, and 5%) were investigated by the VSM technique. As shown in Fig. 1b, the magnetic values of pure MNPs and the composite of MNPs-PF nanoparticles were 50.98, 42.59, 36.71, and 25.76 emu g^−1^, respectively. The decrease in the magnetic value in the composite is due to the polymer coating. The magnetic property of the nanocomposite should be sufficient because a rapid separation was observed by applying an external magnetic field.

One of the techniques for identifying the structure of magnetic nanoparticles is TGA. This technique measures changes in the weight of the sample with temperature. Fig. 1c shows the thermal calorimetry technique for Fe_3_O_4_ nanoparticles. As shown in this figure, weight loss at less than 300 °C is probably related to the evaporation of hydroxyl groups and separation of adsorbed solvents.^6^

According to Fig. 1d, PF has two individual peaks: a peak at 2885, which belongs to C–H stretching vibrations, and a peak at 1250 cm^−1^, which belongs to C–O stretching vibrations.^16^ Peaks at 580, 3400 and 1630 cm^−1^ are due to the presence of iron oxide in the mixture, while the peaks at 1250 and 2885 cm^−1^ are due to the presence of PF in the composite. Moreover, the intensity of the peaks for 5% PF is much higher than for 1% and 2% PF in the composite.^16^

**Fig. 2:**
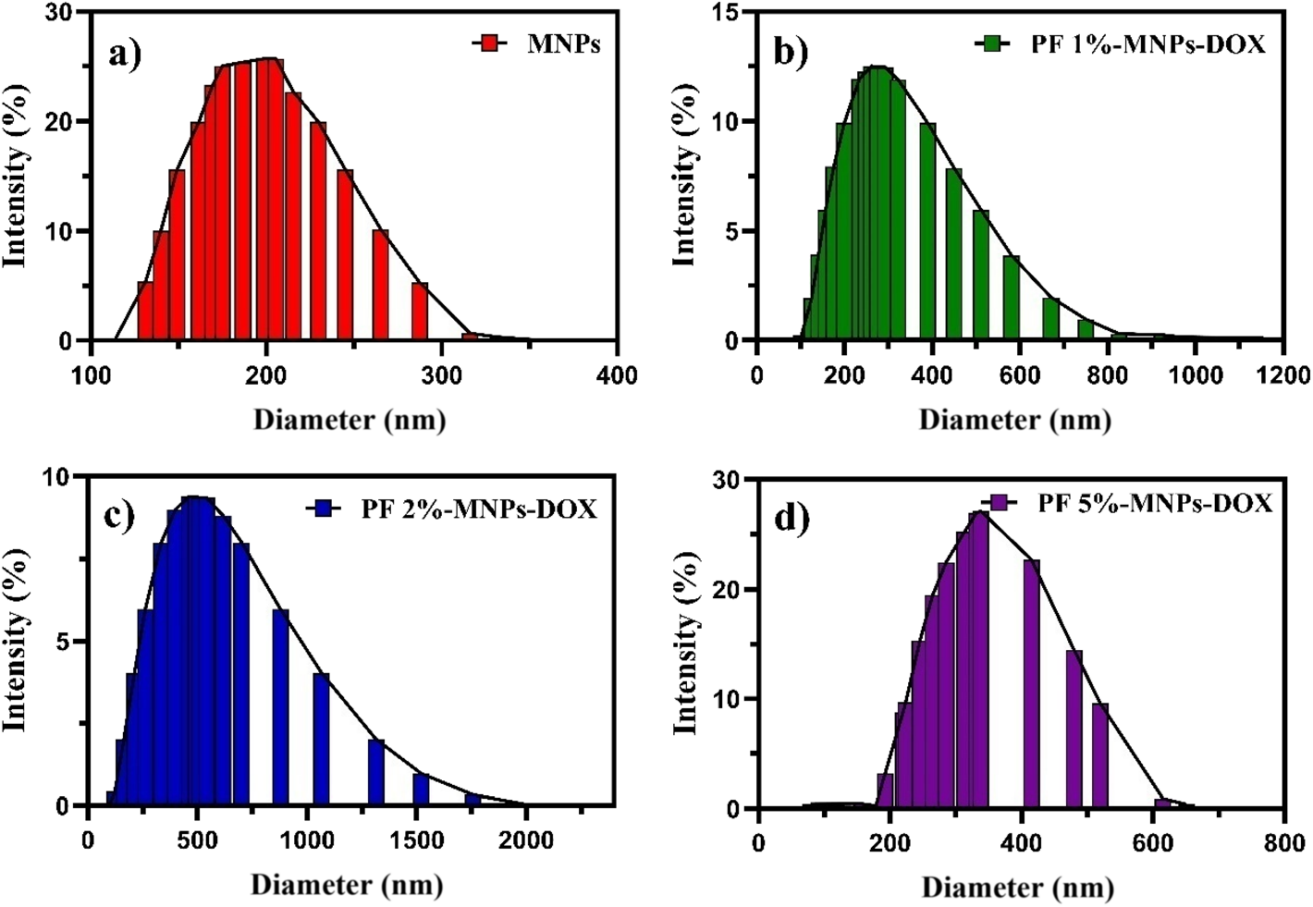
Dynamic light scattering particle size distributions of (a) pure MNPs, (b) PF 1%-MNPs-DOX, (c) PF 2%-MNPs-DOX, and (d) PF 5%-MNPs-DOX.

DLS analyses were performed for nanocomposites of pure MNPs and MNPs coated with DOX-loaded PF (three different concentrations). The polydispersity index (PDI) and average diameter of the MNPs were 189.3 nm and 0.217, respectively (Fig. 2a). After coating with PF, the PDI and average diameter for 1% PF were respectively 264.8 nm and 0.192, while for 2% PF, they were 445.3 nm and 0.440, and 338.9 nm and 0.176 for 5% PF (as shown in Fig. 2b–d), confirming the polymeric coating on the surface of the MNPs.

As the PF concentration increased, the average diameter also increased, indicating an effective coating on the surface of the nanocomposites, except for PF 5%, where the particle size decreased. This may be due to the PF concentration increment and its amphiphilic nature. As the PF concentration increases, the number of hydrophilic groups of the PF chain that have the connectivity to bind to the surface of the MNPs increases. On the other hand, the hydrophobic functional groups in the PF chain come closer to each other, creating an interaction and repulsion, preventing further increase in binding between the PF and the MNPs. In fact, the hydraulic diameter is lower in the 5% PF coating than in the 2% coating. The effects of PF concentration (1, 2, and 5 w%) and pH (7.4 and 5.5) on the zeta potential and particle diameter are shown in Table 1. The results indicate that an increase in PF concentration led to an increase in the average diameter and a diminution in zeta potential, except for 5% PF. This phenomenon can be attributed to the amphiphilic nature of the PF coating on the surface of the MNPs. Moreover, the covering polymer protected the surface charge of the MNPs, which led to a reduction in zeta potential. The zeta potential increased and the average diameter of the particles decreased when the pH was reduced to 5.5. Conversely, the pH increased to 7.4 (due to reducing the zeta potential), which increased the particle size. As a result, the produced nanocarrier has a high potential for delivering DOX to acidic malignant human body tissues and is pH sensitive.

**Table 1:** Particle sizes and zeta potentials of samples with different PF127 contents at various pH values (1 atm and 37 °C)

Sample code	pH	Zeta (mV)	SD (mV)	Particle size (nm)	PDI
MNPs	7.4	−31.5	6.84	189.3	0.217
PF 1%-MNPs-DOX	7.4	−28.3	7.61	264.8	0.192
PF 2%-MNPs-DOX	7.4	−15.1	10.4	445.3	0.440
PF 5%-MNPs-DOX	7.4	−12.3	65.8	338.9	0.176
PF 1%-MNPs-DOX	5.5	−14.54	8.58	243.6	0.205
PF 2%-MNPs-DOX	5.5	−10.58	15.6	437.5	0.305
PF 5%-MNPs-DOX	5.5	13.2	7.76	319.8	0.198

**Fig. 3:**
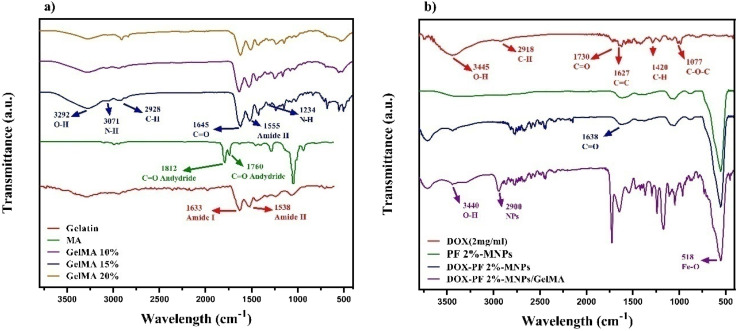
FTIR spectra of (a) gelatin and GelMA (with three different percentages) and (b) DOX, PF 2%-MNPs, DOX-loaded nanoparticles, and DOX-PF 2% MNPs blended on GelMA (15%).

Fig. 3a shows FTIR analysis of gelatin. The peak at 3433 cm^−1^ belongs to the hydrogen bond of water. The peaks in the range from 3287 to 3292 cm^−1^ and 1538 to 1633 cm^−1^ may be associated with amide, while the peaks in the range from 1380 to 1460 cm^−1^ are attributed to symmetric and asymmetric vibrations of the methyl group. Gelatin is a type of protein, and it has amino acids that are connected by amide bonds. Amide bands represent different vibrational modes of peptide bonds. The absorption band of GelMA is located in the amide group region, as shown in Fig. 3a. A peak at 1243 cm^−1^ is due to amide groups and is related to the vibration of the N–H bond. This is partly related to the N–C bond. A peak at 1555 cm^−1^ is also due to amide and is related to the N–H bonds. The peak at 1645 cm^−1^ is also attributed to amide, indicating the vibration of the C=O bonds. The anhydride C=O bands in the crude sample were observed at 1812 and 1760 cm^−1^, and disappeared after dialysis; no peaks corresponding to unreacted monomer/by-products were detected.

The peak at 3433 cm^−1^ represents O–H and N–H stretching vibrations. The peaks in the range from 2800 to 3100 cm^−1^ were attributed to H (C–H) stretching vibrations.^26^ Furthermore, the peaks from the 15% GelMA sample were much more intense than those from the 10% and 20% GelMA concentrations.

The FTIR spectrum of pure DOX has many distinctive peaks. O–H stretching vibrations occur at 3445 cm^−1^, C–H stretching vibrations at 2918 cm^−1^, and C=O bond vibrations at 1730 cm^−1^. Aromatic C–H bending vibrations occur at 1420 cm^−1^, C=C ring vibrations at 1627 cm^−1^, C–O–C bond vibrations at 1077 cm^−1^, and out-of-plane bending vibrations of C–H bonds in the anthracycline chromophore ring are noted at 814 cm^−1^, as shown in Fig. 3b.

The presence of DOX and PF 2%-MNPs in the DOX-PF 2%-MNPs spectrum was also evident. The sharp peaks were observed in the regions of 2885 cm^−1^ and 580 cm^−1^, which are related to aliphatic CH, indicative of MNPs. The peak at 3422 cm^−1^ is characteristic of the OH group, and the peak at 1638 cm^−1^ is associated with the C=O factor group. In the functional group of DOX, a wide peak was observed in the regions of 3436 cm^−1^, and the peak of 1631 cm^−1^ represents the C=O factor group. Finally, in the DOX-PF 2%-MNP/GelMA spectrum, a peak was observed in the region of 3400 cm^−1^, which indicates the presence of DOX, and a sharp peak around 2900 cm^−1^ proves the presence of NPs in the composite. In the blended composite spectrum, the peaks of PF and GelMA, which are related to NH_2_ and OH tensile vibrations, change to higher frequencies, which can indicate hydrogen bonds between the NH_2_/OH group of PF, the NH_2_/OH group of DOX, and or the OH group of GelMA.

**Fig. 4:**
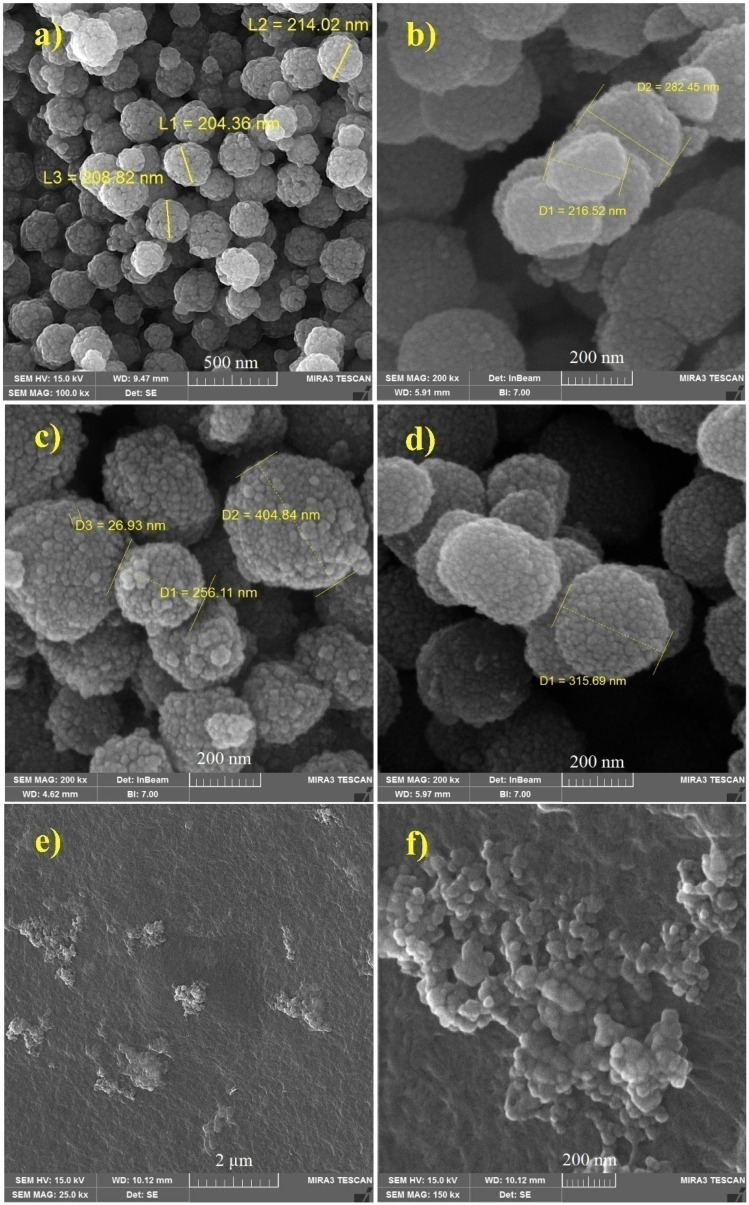
FESEM images of (a) pure MNPs, (b) PF 1% with MNPs, (c) PF 2% with MNPs, (d) PF 5% with MNPs, and (e and f) DOX-loaded PF 2%-MNPs blended on GelMA (15%).

Field Emission Scanning Electron Microscopy (FESEM) was used to examine the surface morphology, size and uniformity of the samples. Fig. 4 shows that the composites have spherical morphology. The nanoparticles were aggregated due to their magnetic properties. The nanocomposites (with Pluronic F127) would be less aggregated than the pure nanoparticles. This may be due to having a larger sized particles in the nanocomposite.^16,18^ According to Fig. 4a–d, the average sizes of the MNPs and the nanocomposite increase with increasing polymer concentration. This indicates the coating influence on the surface of the nanocomposites. The mean diameter size of the MNPs was approximately 208 nm (Fig. 4a), while it respectively increased to 282, 404 and 315 nm in samples coated with PF at concentrations of 1%, 2%, and 5%. According to Fig. 4e and f, PF 2%-MNPs-DOX were completely and uniformly dispersed in the GelMA composite.

### 3.2. GelMA swelling

The swelling test of the GelMA hydrogel was investigated after 48 h. The swelling rate of hydrogels is an important aspect of their medical applications because its changes will affect their surface properties and mobility. Moreover, it affects the solvent's ability to penetrate the hydrogels. The swelling rate in hydrogels is a function of polymer porosity and the interaction of solvent and polymer.^27^ Swelling data of several samples with 10%, 15% and 20% GelMA are illustrated in Table 2 (and Table S1). All samples were exposed to ultraviolet light under identical conditions of 365 nm, irradiation time of 20 min, and room temperature.

**Table 2:** Swelling percentages of the GelMA hydrogels at different concentrations, measured at 24 h (mean ± SD, *n* = 3)

GelMA (%)	*W*_d_ (mg)	*W*_*t*_ (mg)	*W*_*t*=10_ (mg)	SC_*t*_ (%)	SC_*t*=10_ (%)
10	23.39	101.28	85.90	213.2 ± 14.3	165.1 ± 16.6
15	44.70	150.28	118.50	237.0 ± 19.0	165.0 ± 9.2
20	88.60	282.52	234.10	218.9 ± 5.90	164.9 ± 3.6

The swelling rate sharply decreased with increasing the initial concentration of GelMA. In fact, higher GelMA concentrations produced hydrogels with greater density and a higher degree of crosslinking. Moreover, the photoinitiator weight ratio increment led to more dangling functional groups of the polymer per unit volume during the curing process. This would increase the swelling, compaction, and crosslinking and the pore size of the hydrogel would decrease as well.^28^

The swelling behavior of the GelMA-based nanocomposite plays a critical role in controlling the drug-release mechanism. As the hydrogel absorbs water and exhibits a higher swelling ratio, the increased water uptake promotes polymer-network relaxation and enlarges the mesh size of the matrix. This structural loosening facilitates the diffusion of DOX molecules through the hydrogel and results in an accelerated release profile. Therefore, the swelling characteristics are directly correlated with the degradation-mediated drug-release behavior of the composite, and understanding this relationship is essential for predicting the therapeutic performance of the system.

### 3.3. GelMA degradation

The degradation data of various samples with 10, 15 and 20% GelMA are illustrated in Table 3 (and Fig. S2 & Table S2). The amount of photoinitiator and the duration of exposure to ultraviolet light were also considered under constant conditions. The degradation of GelMA hydrogels was monitored at various concentrations over a period of 90 days to evaluate the long-term stability and degradation behavior of the hydrogels. The results showed that the degradation rate would sharply decrease with increasing the initial concentration of GelMA. The destruction rate may be supported by a similar analysis as mentioned for the swelling behavior.

**Table 3:** Degradation of the GelMA hydrogels at different concentrations, measured over 90 days [mean ± SD, *n* = 3 (Fig. S2)]

GelMA (%)	*W*_d_ (mg)	*W*_*t*_ (mg)	Percentage of degradation (%)
10	40.92	14.88	63.5 ± 3.3
15	50.45	16.25	67.8 ± 2.5
20	76.19	45.97	39.6 ± 1.4

The hydrogel's equilibrium water content dropped in proportion to the GelMA concentration. The interconnecting GelMA network chains in the hydrogel were densely packed to create a denser network structure as the GelMA concentration rose. The hydrogel's equilibrium water content fell because of the hydrogel's lower porosity, which also decreased the swelling effect of water molecules on the hydrogel to some degree.

After the degradation of the GelMA hydrogel, the DOX-loaded MNP-PF nanoparticles are gradually released into the surrounding environment. Depending on their size, surface functionality, and concentration, these nanoparticles can undergo different biological fates. They may be internalized by cells and processed through lysosomal pathways, or they can be cleared through the reticuloendothelial system (primarily the liver and spleen). Although MNP-based systems are generally considered biocompatible, potential concerns such as local particle accumulation, oxidative stress, or inflammation at high doses should be taken into account. These considerations are important for the long-term biosafety of MNP-based drug-delivery platforms.

### 3.4. Drug entrapping

**Fig. 5:**
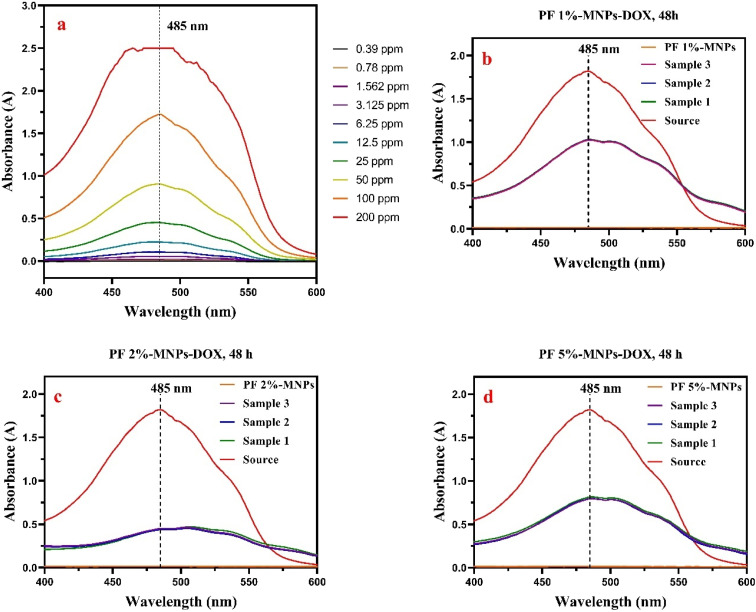
UV-Vis spectra of (a) free DOX, (b) PF 1%-MNPs-DOX, (c) PF 2%-MNPs-DOX, and (d) PF 5%-MNPs-DOX.

Fig. 5 shows UV-Vis spectrometer spectra for determining the successful encapsulation of DOX. As shown in Fig. 5a, the calibration curve of the free DOX based on the serial dilution method was plotted as a function of its absorption intensity at a wavelength of 485 nm. The PF-MNPs and three samples of each percentage of PF in PF-MNPs-DOX nanocomposites were compared to free DOX, as shown in Fig. 5b–d. The results revealed a DOX-related absorbance peak at 485 nm associated with the PF 1%-MNPs-DOX, PF 2%-MNPs-DOX, and PF 5%-MNPs-DOX, confirming the successful DOX encapsulation.

Regarding the remaining drug, it should be noted that DOX release occurs in two stages. The first stage involves an initial burst release due to swelling and diffusion from the hydrogel matrix. The second stage corresponds to the sustained release of the remaining drug, which is closely associated with the progressive degradation of the hydrogel over a 90-day period. Therefore, the remaining drug is gradually released as the hydrogel network breaks down, providing a controlled and extended drug release profile.

As illustrated in Table 4, the entrapment efficiency (EE) and drug loading efficiency (DLE) were maximally found to be 74.73% ± 0.549% and 2.60% ± 0.019% [Table S3 (average of 3 repetitions)] for the 2% PF composite with iron oxide nanoparticles, respectively. The drug loading process was mainly based on the physical adsorption mechanism inside the nanoparticles.^29^ In the controlled release of the drug, the patient does not suddenly receive a high dose of the drug after taking it. In fact, the drug would be released in a suitable dose and this remains constant over time. According to the *in vitro* study, the highest release rate of DOX from the nanocomposite (with PF 2%) was around 74.73% ± 0.549% after 48 h at a pH of 7.4 and 37 °C.

**Table 4:** Drug entrapment efficiency (%) of different nanoparticle samples (mean ± SD, *n* = 3)

PF-MNPs	EE%	DLE%
1%	43.61 ± 0.251	1.52 ± 0.009
2%	74.73 ± 0.549	2.60 ± 0.019
5%	55.87 ± 0.610	1.94 ± 0.022

### 3.5. *In vitro* evaluation of drug release

**Fig. 6:**
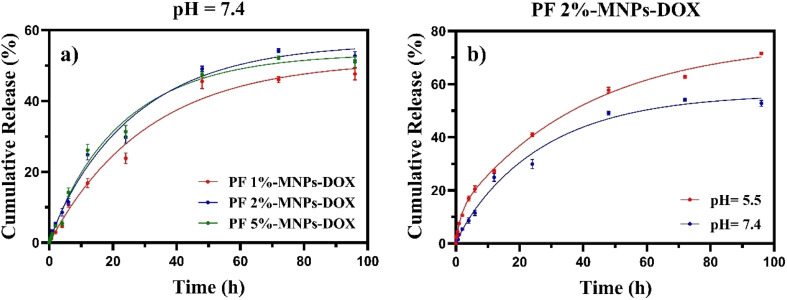
Release patterns of (a) DOX from DOX-loaded MNPs-PF with three different percentages of PF coated on MNPs at pH 7.4 and (b) PF 2%-MNPs-DOX at two different pH levels (7.4 and 5.5).

Three samples of nanoparticles with different concentrations of PF were prepared to investigate the release of DOX from the nanoparticles. The effect of various percentages of PF used in the nanoparticles on the amount of drug release was investigated. The release behavior of DOX from the nanoparticles was evaluated at a pH of 7.4. The variation in release demonstrated that the DOX-loaded PF-MNPs composite functioned as a PF concentration-responsive delivery system influenced by ingredient concentration. Fig. 6a shows the DOX release profile from nanoparticles at three different ratios of PF. The DOX release increased with increasing PF percentage. Consequently, the release of DOX was respectively enhanced to 47.63%, 52.74%, and 50.99% by 1%, 2%, and 5% of PF at pH of 7.4 over a period of four days. The minimal release of DOX at a pH of 7.4, which corresponds to the pH of blood and normal tissues, contributes to reducing the adverse side effects associated with DOX as a powerful anti-cancer agent. The cumulative DOX release of DOX-loaded MNPS blended with 1%, 2%, and 5% of PF had an analogous pattern before 10 h.

It is well established that the extracellular pH of cancerous tissue is lower than that of healthy tissue. Several researchers have attempted to create pH-responsive delivery carriers to take advantage of this disparity. The profile of DOX release under different pH conditions (7.4 and 5.5) was used only for PF 2%-MNPs-DOX due to its higher DLC percentage. Correspondingly, as illustrated in Fig. 6b, the *in vitro* release of DOX from the PF 2%-MNPs-DOX showed sustained release patterns under acidic (pH = 5.5) and neutral (pH = 7.4) conditions. The drug release for a pH of 5.5 was much higher than that for a pH of 7.4. After 24 h of release, more than 42% of the total drug was liberated at a pH of 5.4. Furthermore, the DOX release from the PF 2%-MNPs was pH sensitive, showing a slower release rate in neutral conditions than in acidic ones. These results are consistent with the recent findings regarding stronger hydrogen bonding interactions at neutral pH and the higher solubility of DOX in acidic environments.^30,31^

### 3.6. Drug release modeling

**Fig. 7:**
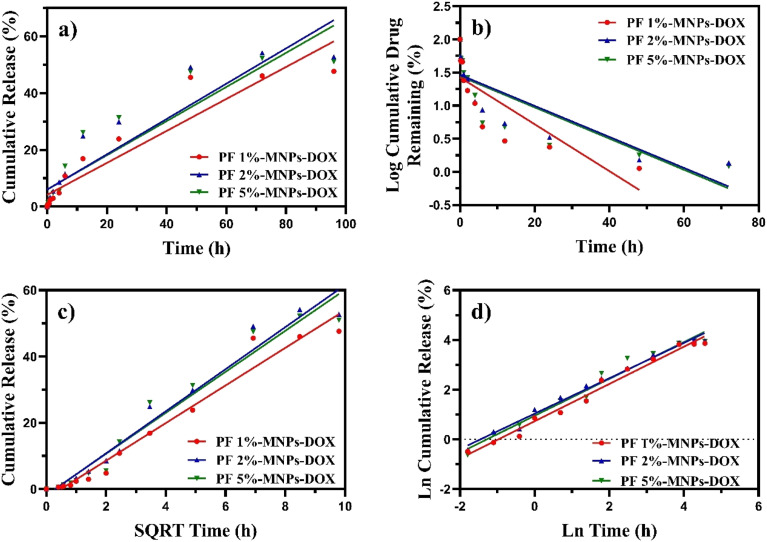
Various models for DOX release at pH 7.4 for 2% Pluronic nanocomposite: (a) zero-order, (b) first-order, (c) Higuchi, and (d) Korsmeyer–Peppas models.

Herein, the drug release mechanism was studied through the kinetic models (zero-order, first-order, Higuchi, Korsmeyer–Peppas). This examination involved fitting the experimental release data at a pH of 7.4 using eqn (6)–(9), associated with graphs of Fig. 7. The exponent value in the Korsmeyer–Peppas model shows that the release mechanism is governed by Fick's law (diffusion) when *n* ≤ 0.34, while it follows case II transport, involving the swelling and relaxation of the polymer matrix when *n* ≥ 0.85. It indicates an anomalous mechanism, combining Fickian diffusion with polymer matrix erosion when 0.34 < *n* < 0.85.

The Korsmeyer–Peppas model was selected to describe the drug release mechanism using the GelMA-based hydrogels due to its suitability for hydrophilic polymer systems, which drug release occurs *via* a combination of diffusion and polymer swelling. This model has been widely validated in similar hydrogel-based drug delivery studies, providing insights into the release mechanism through the diffusional exponent, *n*.^32^

As shown in Table 5, the Korsmeyer–Peppas model has the highest *R*^2^ (≈1) compared with the other models for the 2% PF nanocomposite (which had the maximum EE% and DLE%). Furthermore, its power value (*n*) was calculated as 0.7497, 0.7120, and 0.7418 for PF 1%, 2%, and 5%, respectively. This indicates that the release mechanism follows the anomalous mechanism.^33^ In other words, the mechanism is based more on polymer matrix erosion than on Fickian transport.

**Table 5:** Correlation coefficients of the fit of each model for nanocarriers at pH = 7.4 and pH = 5.5

Composite	Zero order	First order	Higuchi	Korsmeyer–Peppas	
	*R* ^2^	*R* ^2^	*R* ^2^	*R* ^2^	*n*
**pH = 7.4**					
PF 1%-MNPs-DOX	0.8901	0.6761	0.9675	0.9827	0.7497
PF 2%-MNPs-DOX	0.8698	0.7206	0.9710	0.9860	0.7120
PF 5%-MNPs-DOX	0.8481	0.6885	0.9620	0.9745	0.7418

**pH = 5.5**					
PF 2%-MNPs-DOX	0.8934	0.7812	0.9912	0.9876	0.5649

**Fig. 8:**
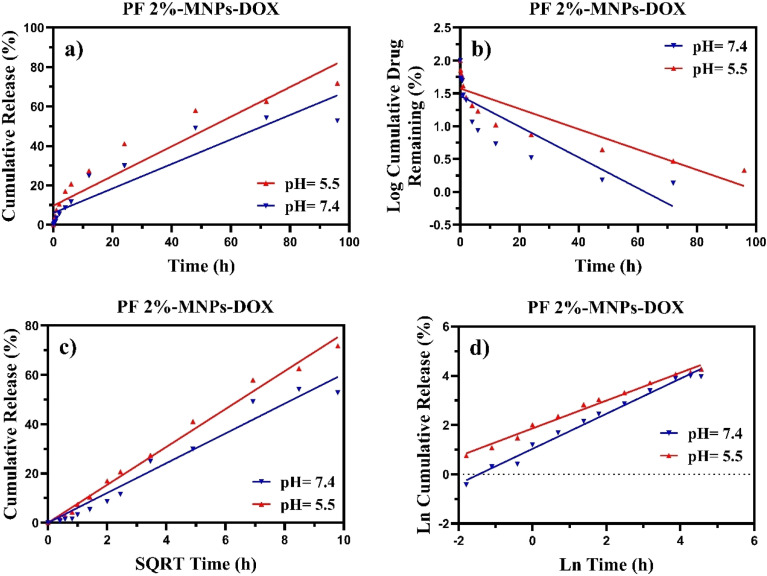
*In vitro* release profile of PF 2%-MNPs-DOX under different pH levels (7.4 and 5.5) at 37 °C: (a) zero-order, (b) first-order, (c) Higuchi, and (d) Korsmeyer–Peppas models.

The final data revealed better DOX release under acidic conditions, which can be attributed to the pH sensitivity of 2% PF. Additionally, the results confirmed the long-term release of DOX from the nanocomposites, which is crucial for reducing side effects and boosting drug accumulation in tumour tissues, and also demonstrated the controlled release capabilities of PF 2%-MNPs-DOX in acidic conditions. To investigate the stability of the drug loaded in the PF 2%-MNPs-DOX nanocarrier under acidic (pH = 5.5) conditions, DOX-release data were measured using four different kinetic models involving zero-order, first-order, Higuchi, and Korsmeyer–Peppas models. They were evaluated with their results under neutral (pH = 7.4) conditions, as shown in Fig. 8.

As illustrated in Table 5, the power value (*n*) for PF 2%-MNPs under acidic (pH = 5.5) conditions was 0.5649, which indicates that the release mechanism combines Fickian diffusion with polymer matrix erosion. Compared to the calculated power under neutral (pH = 7.4) conditions, which is more polymer erosion than Fickian diffusion, drug release in an acidic environment follows both mechanisms, resulting in more stable drug release.

### 3.7. Cell cytotoxicity

**Fig. 9:**
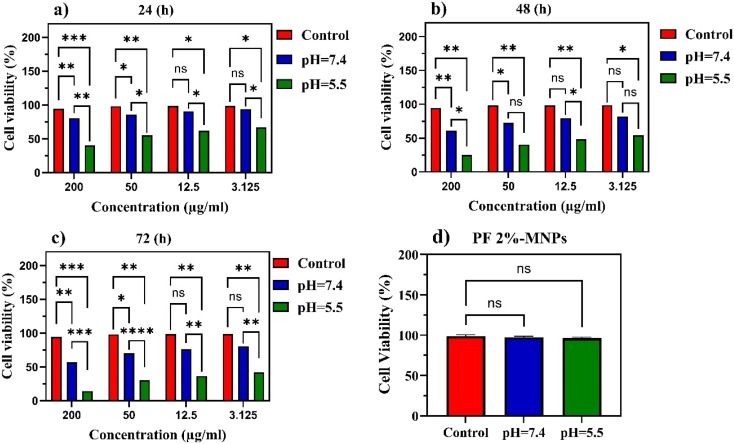
Cell viability of PF 2%-MNPs and DMED media over the MCF-7 cell line at different pH values: (a) 24 h, (b) 48 h and (c) 72 h for four different concentrations of DOX, and (d) carrier without DOX. Notes: *denotes *P* < 0.05, **denotes *P* < 0.01, ***denotes *P* < 0.001, and ****denotes *P* < 0.0001.

To evaluate the pharmacological activity and *in vitro* biocompatibility of the nanocarrier, an MTT assay was performed. The PF 2%-MNPs-DOX nanocarrier was chosen for this investigation due to its high DLC and EE (2.59 and 74.72%, respectively). The assay was carried out by increasing concentrations of DOX in the PF 2%-MNPs-DOX nanocarrier. Fig. 9 shows, the *in vitro* viability of MCF-7 cancer cells after 24, 48 and 72 h treatment with DOX formulated in MNPs PF 2%, and carrier without DOX.

As shown in Fig. 9a, a few general outcomes were immediately identified. The DOX carrier exhibited greater cytotoxicity under acidic (pH = 5.5) than under neutral (pH = 7.4) conditions, especially at the highest concentration (200 µg ml^−1^). The cellular viability of the DOX carrier was approximately 40%, while it was more than 80% in the other pHs (*P* < 0.01). The results illustrated significant time and concentration-dependent cell growth inhibition for the acidic (pH = 5.5) environments. A similar trend of cytotoxicity was also observed at 48 and 72 h. As shown in Fig. 9d, the data exhibited nontoxic effects after 24 h for both pHs. Consequently, it could be anticipated that *in vivo* PF-covered MNPs would be far less toxic than the other vehicles. If so, then the administration of DOX-loaded PF-MNP nanocarriers at higher drug doses would be possible.

## 4. Conclusions

Herein, we aimed to synthesize a suitable system for loading and releasing doxorubicin as a cancer treatment medicine. For this purpose, a nanocomposite based on Pluronic F127 copolymer/iron oxide–GelMA nanoparticles was successfully synthesized and characterized. Several parameters, such as various polymer concentrations, pH and drug loading time, were investigated. Iron oxide nanoparticles were synthesized by the hydrothermal method and then composited with polymer by the ultrasonication process. Several tests confirmed nanocomposite formation and good porosity (due to the presence of GelMA in its structure). The hydrogel caused swelling and degradation, which decreased with increasing initial concentrations of GelMA. The doxorubicin release kinetics showed a non-Fickian permeation mechanism at a pH similar to that of blood and at ambient temperature. The results indicate that the PF-MNP/GelMA composite provides a stable and controllable platform for DOX delivery. The combination of chemical interactions and physical entrapment ensures high loading efficiency, while the gradual hydrogel degradation under physiological conditions supports prolonged and predictable drug release. Although the current study provides a comprehensive characterisation and *in vitro* evaluation of the Pluronic PF/GelMA/iron oxide nanoparticle system, certain aspects of biocompatibility and intracellular performance were not investigated. Specifically, hemolysis assays, protein adsorption studies, immune response evaluations, as well as cellular internalisation and intracellular drug release measurements, were not performed. These represent important factors for clinical translation, and future studies will aim to systematically address these parameters to provide a more complete assessment of the nanocarrier's safety and therapeutic efficacy.

## Conflicts of interest

There is no conflict of interests on this research.

## Supplementary Material

NA-008-D5NA00776C-s001

## Data Availability

Data underlying the results presented in this paper are not publicly available at this time but may be obtained from the authors upon reasonable request. Supplementary information (SI) is available. See DOI: https://doi.org/10.1039/d5na00776c.
