# Supplementary material for: Synthesis of Pluronic F127 copolymer/iron oxide–GelMA nanocomposite for doxorubicin drug delivery
Source: Nanoscale Adv. 2026 Jan 2;8(5):1611–25. doi: 10.1039/d5na00776c (PMC12867036; doi:10.1039/d5na00776c)
Supplement: NA-008-D5NA00776C-s001 [file NA-008-D5NA00776C-s001.pdf]

Supplementary Information (SI) for Nanoscale Advances.
This journal is © The Royal Society of Chemistry 2026

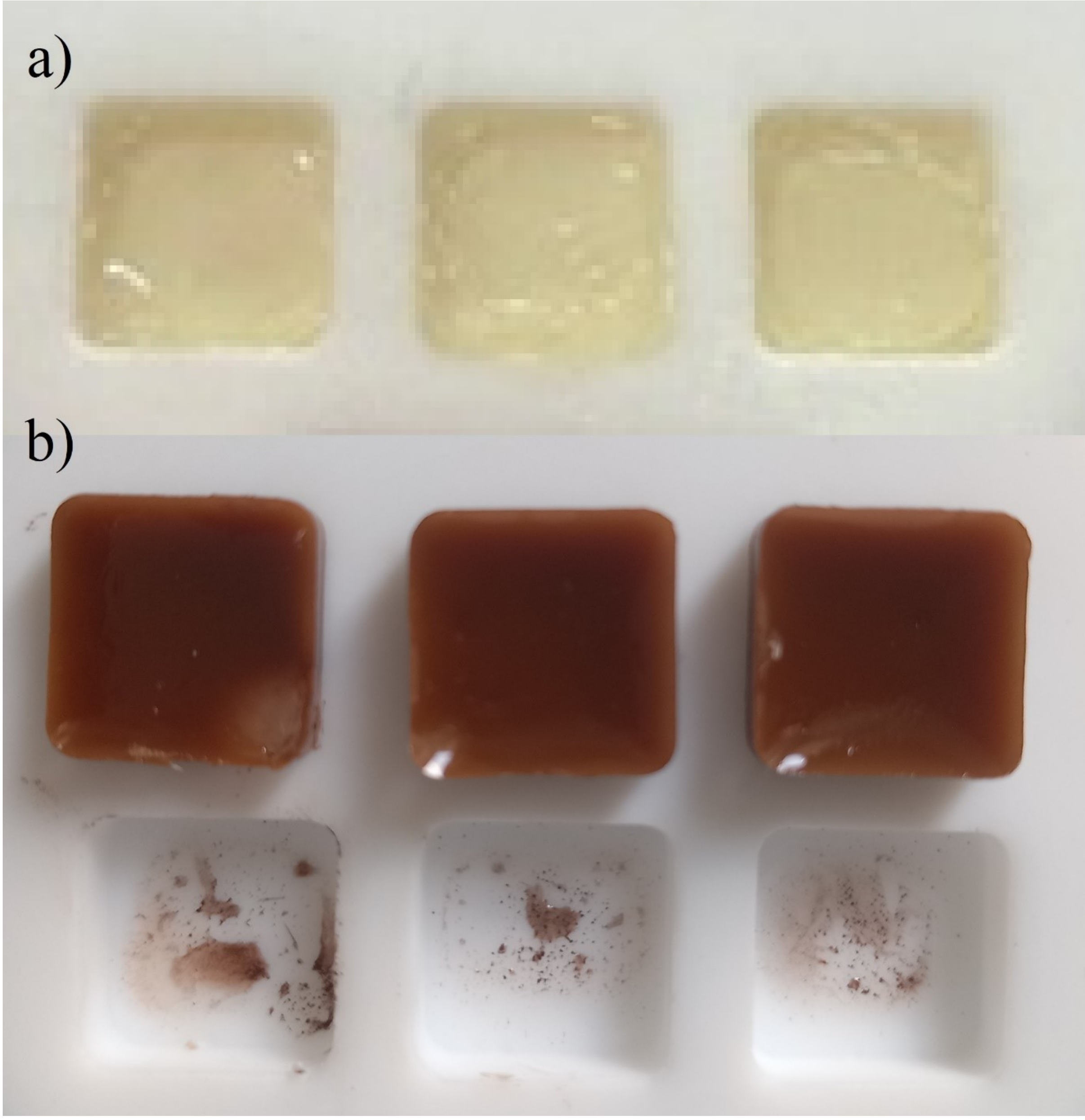

Fig. S1. Photograph representation of the mould used for GelMA hydrogel casting and the corresponding sample preparation steps: a) GelMA, and b) PF 2% MNPS-DOX

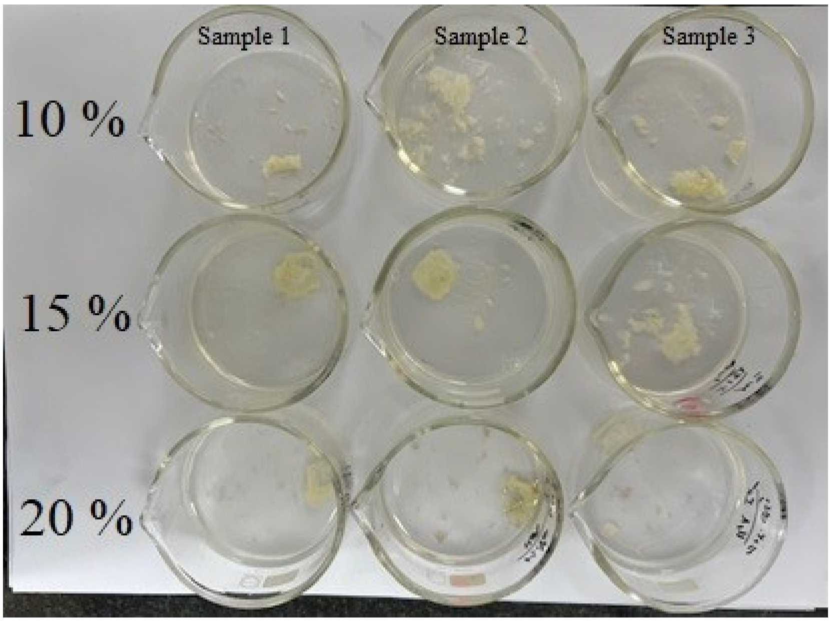

Fig. S2. Photographs of GelMA hydrogels during daily degradation in PBS at 37 °C

Table S1. Mean ± SD of swelling percentage of GelMA hydrogels at various concentrations (n = 3)

GelMA (%)		W_d (mg)	W_t (gm)	$W_{t=10}$ (mg)	SC_t (%)	$SC_{t=10}$ (%)	Mean±SD_t	Mean±$SD_{t=10}$
10	Sample 1	32.47	102.48	87.26	215.6	168.6	213.2 ± 14.3	165.1 ± 16.6
	Sample 2	34.12	101.6	84.60	197.8	147.9		
	Sample 3	30.58	99.75	85.85	226.1	180.7		
15	Sample 1	46.287	148.32	121.3	220.3	161.8	237.0 ± 19.0	165.0 ± 9.2
	Sample 2	42.57	152.12	117.36	257.6	175.8		
	Sample 3	45.23	150.7	116.85	233.1	158.3		
20	Sample 1	88.59	283.48	235.65	220.0	165.8	218.9 ± 5.9	164.9 ± 3.6
	Sample 2	89.97	281.26	233.68	212.6	159.7		
	Sample 3	87.25	282.82	232.98	224.2	167.1		

Table S2. Mean ± SD of degradation percentage of GelMA hydrogels at various concentrations (n = 3)

GelMA (%)		W_d (mg)	W_t (gm)	Percentage of degradation (%)	Mean±SD
10	Sample 1	41.36	13.84	66.5	63.5 ± 3.3
	Sample 2	41.84	14.86	64.5	
	Sample 3	39.56	15.93	59.7	
15	Sample 1	51.73	15.24	70.5	67.8 ± 2.5
	Sample 2	49.15	16.7	66.0	
	Sample 3	50.48	16.81	66.7	
20	Sample 1	75.43	44.36	41.2	39.6 ± 1.4
	Sample 2	76.29	46.87	38.5	
	Sample 3	76.84	46.68	39.2	

Table S3. Mean ± SD of drug entrapment efficiency of PF-MNPs at various concentrations (n = 3)

PF-MNPs (%)		W_{DOX} (mg)	W_{Source} (gm)	EE (%)	DLE (%)
1	Sample 1	0.0585	0.1043	43.61±0.251	1.52±0.009
	Sample 2	0.0588	0.1043		
	Sample 3	0.0591	0.1043		
2	Sample 1	0.0264	0.1043	74.73±0.549	2.60±0.019
	Sample 2	0.0257	0.1043		
	Sample 3	0.0269	0.1043		
5	Sample 1	0.0456	0.1043	74.73±0.549	1.94±0.022
	Sample 2	0.0468	0.1043		
	Sample 3	0.0457	0.1043		
